# Pediatric inflammatory bowel disease and cancer

**DOI:** 10.3389/fimmu.2025.1624177

**Published:** 2025-08-27

**Authors:** Jia-Jing Zheng, Zhi-Fei Wu, Yi-Bing Hu

**Affiliations:** ^1^ Department of Pediatrics, Beilun District People’s Hospital, Ningbo, Zhejiang, China; ^2^ Department of Gastroenterology, Affiliated Jinhua Hospital, Zhejiang University School of Medicine, Jinhua, Zhejiang, China

**Keywords:** pediatric, inflammatory bowel disease, cancer, inflammation, immunosuppressive

## Abstract

**Background:**

Children with inflammatory bowel disease (IBD) have a higher risk of cancer due to prolonged exposure to chronic inflammation and immunosuppressive therapies.

**Methods:**

A comprehensive review of extant literature was performed. Findings: The cancer landscape in pediatric IBD is complex, with colorectal cancer, small intestine cancer, lymphoma, cholangiocarcinoma/hepatocellular carcinoma, and skin cancer being predominant concerns. The underlying pathogenic mechanisms involve genomic instability induced by chronic inflammation, carcinogenic effects of immunosuppressants, and environmental factors (e.g., high-fat diet and air pollution). Effective cancer surveillance is crucial in mitigating risk. Strategies include early endoscopic monitoring for high-risk populations, routine dermatological assessments, and clinical monitoring for tumor-related symptoms.

**Conclusion:**

This review synthesizes current evidence on the epidemiological characteristics, pathogenic mechanisms, and clinical management strategies for IBD-related malignancies in children. An in-depth characterization of the mechanisms by which pediatric IBD contributes to tumorigenesis is essential for developing surveillance protocols and advancing research to reduce tumor-associated morbidity.

## Introduction

Inflammatory bowel disease (IBD) refers to a group of chronic, immune-mediated inflammatory disorders, primarily Crohn’s disease (CD) and ulcerative colitis (UC), which can affect multiple organ systems and tissues in the body (1, 2). Despite the advances in management, IBD remains incurable, and the disease course is marked by recurrent episodes of remission and relapse (3). The persistent or progressive inflammation associated with IBD can lead to various complications beyond the gastrointestinal tract, with malignancy being a major concern (4). The incidence of pediatric IBD has shown a steady increase in recent years, and younger age at onset is associated with a heavier disease burden (5). Consequently, the risk of malignancy in pediatric IBD patients is a major concern. Compared to adults with IBD, children are exposed to chronic inflammation for a longer duration, predisposing them to an increased risk of tumor development (6). In addition, pediatric patients often require prolonged and more intensive treatment regimens, potentially contributing to carcinogenesis. Pediatric IBD-associated malignancies include gastrointestinal malignancies (such as colorectal tumors, small intestinal tumors, and cholangiocarcinoma) and extraintestinal malignancies (such as lymphoma and skin cancer) (7–9). The present review aims to enhance the awareness among clinicians, particularly pediatric specialists, regarding the diagnosis and management of malignancies in children with IBD. It systematically summarizes the epidemiological characteristics and potential pathogenic mechanisms underlying IBD-associated malignancies in pediatric patients. A comprehensive overview of preventive and therapeutic strategies is also provided to facilitate clinical decision-making and improve patient outcomes.

## Cancer risk in pediatric patients with IBD

### Colorectal cancer

#### Epidemiology

**Figure 1:**
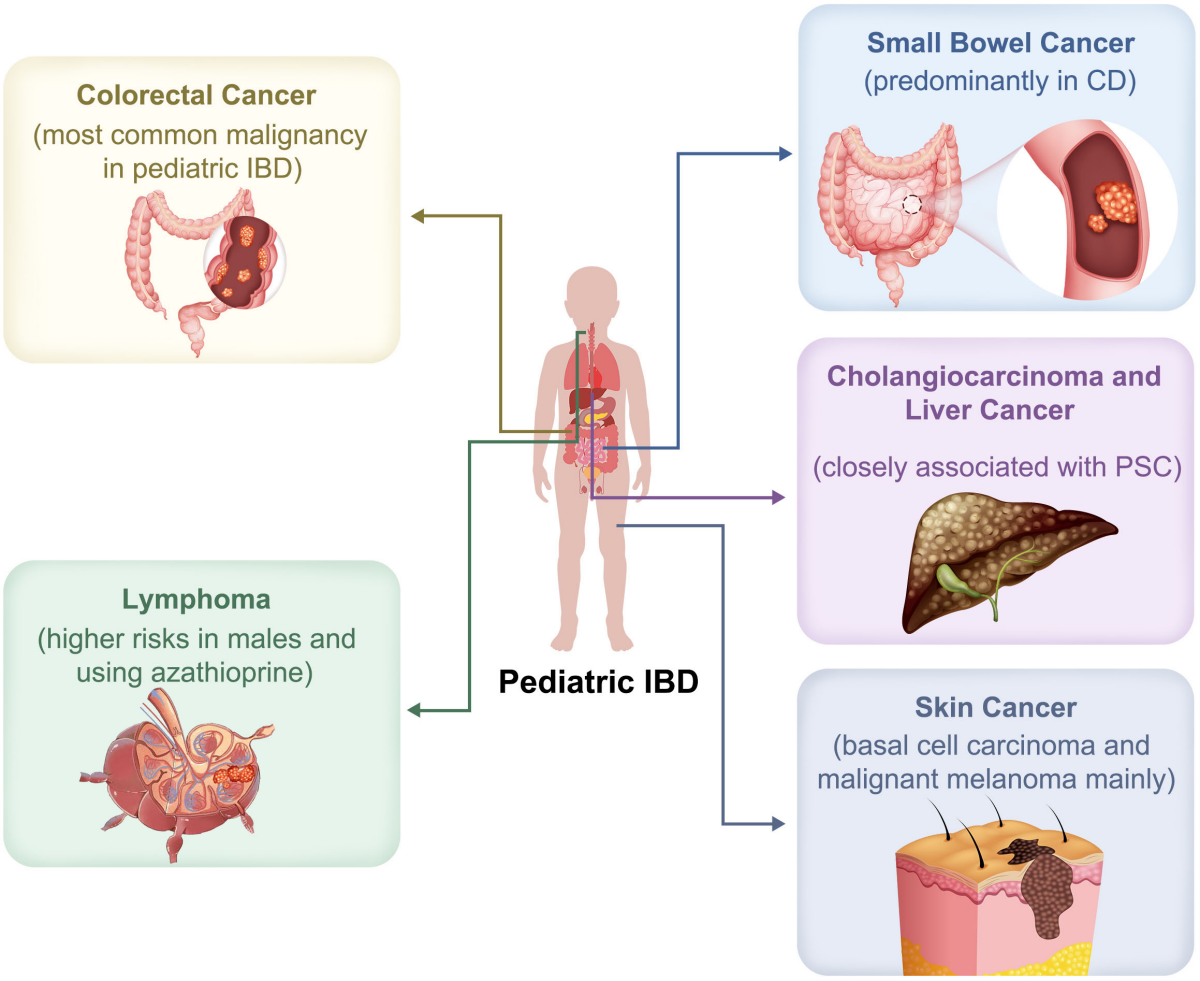
Spectrum of malignancies associated with pediatric inflammatory bowel disease (IBD). Pediatric IBD is associated with an increased risk of various malignancies. Colorectal cancer is the most common. Small bowel cancer occurs predominantly in Crohn’s disease (CD). Lymphoma risk is especially increased in males and those receiving azathioprine. Cholangiocarcinoma and liver cancer are closely linked to primary sclerosing cholangitis (PSC). Skin cancers associated with pediatric IBD include basal cell carcinoma and malignant melanoma.

Colorectal cancer (CRC) is the most common malignancy in pediatric IBD, mirroring trends observed in adults, where IBD confers a 1.40–1.70-fold higher risk due to chronic inflammation (10) (**Figure 1**). A meta-analysis of 20 studies (40,547 patients, 1965–2008) reported CRC as the most frequent malignancy in CD, with an incidence rate of 0.50 per 1,000 person-years—2–3 times higher than in the general population and occurring ~20 years earlier (mean age: 51.5 years), though still lower than in UC (11). There is a relative paucity of data on children in this regard.

#### Risk factors

A Swedish nationwide cohort study of 9,405 pediatric-onset IBD patients (1964–2014) found a markedly elevated gastrointestinal cancer risk (HR: 18.00, 95% CI: 14.40–22.70), particularly CRC (HR: 33.30 for UC, 5.08 for CD), which did not decline over time (12). Long-standing colitis and coexisting primary sclerosing cholangitis (PSC) were key risk factors (12). In a follow-up study, pediatric-onset UC was associated with a significantly higher CRC risk (adjusted HR [aHR]: 37.00 [95% CI: 25.10–54.40]) compared to adult-onset UC (HR: 1.32–1.88) (13). Similar findings have been reported for pediatric-onset CD (14). A Danish–Swedish registry study (1969–2017) involving 6,937 CD patients and 8,514 UC patients (<18 years) showed CRC risk ratios of 6.46 (95% CI: 3.95–10.60) in CD and 32.50 (95% CI: 23.00–45.90) in UC. Risk was highest in colonic CD (L2) (HR: 17.90) and extensive UC (E3/E4) (HR: 36.30, 95% CI: 22.80–57.80), especially with comorbid PSC or younger IBD onset. However, the absence of endoscopic and laboratory data and small CRC case numbers limit these findings (15). Another large cohort study (Pediatric PSC Consortium; n = 509) assessed CRC/dysplasia in pediatric PSC-UC or unclassified IBD (IBD-U) patients (16). The incidence was 2.80 CRC/dysplasia cases per 1,000 person-years (cumulative probability: 0.80% at 5 years and 4.80% at 10 years). Eight patients (1.60%) developed CRC or dysplasia; five were detected *via* surveillance colonoscopy, and three post-colectomy due to refractory colitis. Half of these patients had very early-onset IBD (VEO-IBD, <6 years), with a significantly higher incidence (5.80 *vs.* 1.80 per 1,000 person-years, HR: 3.40), underscoring the need for intensified CRC surveillance in this subgroup.

#### Onset time and cancer types

A Korean retrospective study (2000–2020) of 443 pediatric IBD patients reported four CRC cases (all male) (incidence: 1.29 per 1,000 person-years) (17). Median age at IBD and CBC diagnoses were 11.50 years and 18.50 years, respectively. Most tumors arose in the sigmoid colon (3/4), with histological types including adenocarcinoma (n = 2), mucinous adenocarcinoma (n = 1), and signet-ring cell carcinoma (n = 1). All patients had received azathioprine (AZA, median: 2.27 years); one also received a TNF-α inhibitor. One patient developed peritoneal metastasis and died post-chemotherapy, while the remaining three had early-stage CRC managed surgically. The authors noted that early CRC onset may reflect prolonged inflammation and disease duration, though small sample size and lack of a control group limit conclusions.

### Small bowel cancer

#### Epidemiology

Small bowel cancer is a rare but significant complication of IBD, predominantly affecting patients with CD (**Figure 1**). The exact etiology remains unclear (18). Laukoetter et al. (11) reported it as the second most common CD-associated malignancy (0.30/1,000 person-years). However, the true incidence may be underestimated due to subclinical presentation and limited access to small bowel imaging. A Danish–Swedish cohort (1969–2017) showed a markedly elevated risk in CD (aHR: 9.09) and mortality (aHR: 6.59), while UC patients had a lower, but still increased risk (aHR: 1.85 for incidence; 1.57 for mortality) (19). Limited data analysis exists regarding children.

#### Risk factors and cancer types

Among the CD patients, adenocarcinoma predominated (aHR: 15.80), especially in the ileum and jejunum (50% of cases), followed by neuroendocrine tumors (aHR: 5.51) and sarcomas (aHR: 4.04). Early-onset CD was a key risk factor. A meta-analysis of national registries from Sweden, Denmark, and Finland reported a significantly higher risk in pediatric-onset IBD (relative risk [RR]: 16.20), though the absolute incidence remained low (0.10/1,000 person-years) (20). Subgroup analyses were limited by small sample size and a focus on high-income Western countries. In a French cohort (n = 698), nine malignancies were reported, including one incidentally-detected small bowel carcinoid tumor following pathological examination of the appendectomy specimen (21). A 23-year study in Denmark and Finland (n=6,689) found an elevated standardized incidence ratio (SIR) for small bowel cancer (SIR: 21.80), especially in CD (SIR: 32.60); the increased risk in UC (SIR: 13.30) was not statistically significant. Only three small bowel cancer cases were reported, limiting broader conclusions (22).

### Lymphoma

#### Epidemiology

Lymphoma, a heterogeneous group of lymphoproliferative disorders, can be classified into subtypes based on lymphocyte origin, clinical presentation, and prognosis (23). Available evidence suggests a higher risk of lymphoma in IBD patients, particularly those with CD, likely due to persistent, uncontrolled inflammation (**Figure 1**) (24, 25). A meta-analysis reported a significantly elevated risk of hematologic malignancies in pediatric IBD (RR: 3.10 [95% CI: 1.88–5.10]) compared to the general pediatric population (20), particularly in thiopurine-exposed patients and males (RR: 3.23), while the increased risk in females was not statistically significant (RR: 2.45). However, the findings were limited by a low proportion of thiopurine users and a Western-centric population. A nationwide cohort study in Israel (n = 3,944) reported four non-Hodgkin lymphoma (NHL) cases in pediatric IBD patients (incidence: 1.69 per 10,000 person-years), yielding an RR of 4.40 (95% CI: 1.00–19.60; *p* = 0.06) (26). CD patients had a significantly elevated risk (RR: 10.00, 95% CI: 1.03–100, *p* = 0.04), whereas UC patients did not. Overall, data pertaining to the pediatric population remains scarce.

#### Risk factors and cancer types

Thiopurine monotherapy or combination therapy with anti-TNFα agents was not found to be significantly associated with NHL risk. No hepatosplenic T-cell lymphoma (HSTCL) cases were reported. In contrast, a retrospective multicenter study by the Porto Pediatric IBD Working Group (2006–2011) across 20 European countries and Israel identified 18 malignancy cases in 44 pediatric IBD cases—67% with CD and 22% with UC (27). Hematologic malignancies predominated (n = 12), including HSTCL (three cases), Epstein-Barr virus (EBV)-associated lymphoma (three cases), Hodgkin lymphoma (four cases), and leukemia (two cases). Six cases were linked to long-term thiopurine use (>12 months), suggesting a potential therapy-related risk. Differences in study design and underreporting may explain inconsistencies with the previous Israeli study (27).

### Cholangiocarcinoma and liver cancer

#### Epidemiology

Cholangiocarcinoma is a highly lethal malignancy with limited treatment options (28). Kaj-Carbaidwala et al. (29) investigated the timeline and clinical significance of cholangiocarcinoma development in pediatric-onset PSC patients with IBD. Out of 21 patients who developed cholangiocarcinoma, 38% were diagnosed within two years of the second diagnosis (PSC or IBD), and 75% within 14 years (median: 6.95 years). Nearly half (47%) were diagnosed between the ages of 14 and 25, highlighting the need for enhanced screening during the transition to adult care. However, these findings were partly based on retrospective case reports, warranting prospective validation.

#### Risk factors and cancer types

A Danish–Finnish case-control study (n = 6,689) identified seven cholangiocarcinoma cases among pediatric IBD patients—six in UC (mostly pancolitis), and one in CD with ileocecal disease (30). PSC was identified as a significant risk factor, present in 25% of cholangiocarcinoma cases versus 3.6% in controls. Use of immunosuppressants or biologics was not associated with increased risk. According to the International Agency for Research on Cancer (IARC), liver cancer remains a major global health burden (31). In pediatric-onset IBD, the relative risk of liver cancer is strikingly elevated (RR: 55.45 [95% CI: 19.59–156.99]), surpassing that of CRC (pRR = 20.29) and small intestinal cancer (pRR = 16.20), likely due to its association with PSC. Despite the low absolute incidence of liver cancer, the extraordinarily high RR underscores the need for close surveillance. However, a notable limitation of the study was the lack of distinction between hepatocellular carcinoma and cholangiocarcinoma subtypes, highlighting the need for further research to elucidate the specific risks and mechanisms involved (**Figure 1**) (20).

### Skin cancer

#### Epidemiology

Skin involvement in IBD, presenting as extra-intestinal manifestations (EIM), has been linked to an increased risk of skin cancers, such as malignant melanoma and non-melanoma skin cancer (**Figure 1**) (32). A French study of 698 pediatric-onset IBD patients reported a 1.30% cancer incidence rate, in which two cases of basal cell carcinoma (BCC), which were primarily located on the abdomen and face, required local excision (21).

#### Risk factors and cancer types

A Danish–Finnish study found a 4.20-fold higher risk of skin cancer in pediatric IBD patients compared to the general population (SIR: 2.40–6.70), with significantly increased risk in CD (SIR: 6.70), but not UC (SIR: 2.20) (22). Of 17 total skin cancer cases, 10 were melanomas—predominantly in CD (n=7)—and 7 were BCCs (5 in CD, 2 in UC). A nationwide Danish cohort further confirmed elevated risks for both melanoma (HR: 2.01 [95% CI: 1.19–3.42]) and non-melanoma skin cancer (HR: 2.21 [95% CI: 1.49–3.28]) in pediatric-onset IBD (33). No association was observed with IBD subtype, thiopurine or TNF-α therapy, PSC, or history of colectomy.

## Potential mechanisms of tumor risk

### Chronic inflammation-driven carcinogenesis

**Figure 2:**
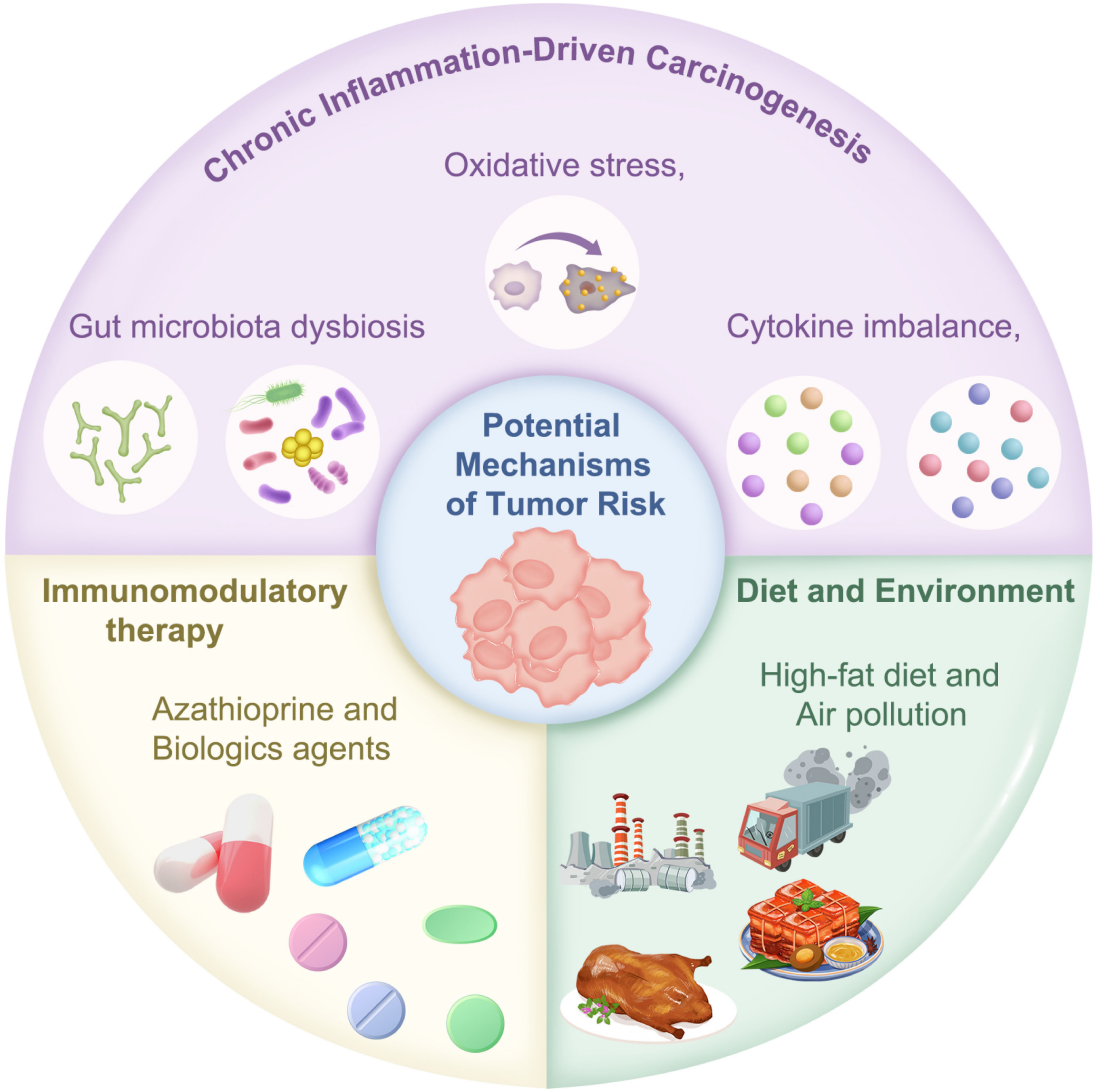
Potential mechanisms underlying tumor risk in pediatric inflammatory bowel disease (IBD). The pathogenesis of cancer in IBD involves genetic variants induced by chronic inflammation, carcinogenic effects of immunosuppressive agents, and environmental factors.

Chronic inflammation promotes carcinogenesis by inducing variants and epigenetic alterations in key regulatory genes, disrupting normal cellular growth control (**Figure 2**) (34). Many basic experiments have corroborated these results. Chronic inflammation also drives oxidative stress and excessive lipid peroxidation, leading to elevated oxidase activity and increased production of reactive oxygen species (ROS) and reactive nitrogen species (RNS). These reactive intermediates cause DNA and mitochondrial damage, impair repair pathways, and inactivate tumor suppressor genes, thereby facilitating tumor development (35).

Inflammatory cytokines contribute to cancer development by inhibiting apoptosis, inducing angiogenesis, and disrupting normal inflammatory signaling (36). Chronic inflammation is marked by immune cell infiltration, which drives cytokine release and stress protein production, forming a microenvironment conducive to cancer development (37–39).

Key inflammatory mediators include proteases, arachidonic acid, cyclooxygenase, IL-4, IL-7, NF-κB, TNF-α, IFN-α, IL-1β, IL-8, and coagulation factors (40–42). Major stress proteins involved are heat shock proteins (HSPs) and glucose-regulated proteins (GRPs). Together, these molecules support tumor cell proliferation, survival, and metastasis, underpinning cancer initiation and progression (43–45).

The tumor suppressor gene p53 plays a central role in colitis-associated cancer (CAC), with early dysplastic changes commonly detected in colitis biopsies (46). These findings have been corroborated by numerous clinical studies. A meta-analysis of 19 studies by Lu et al. (47) reported a significantly higher p53 expression in UC patients compared to normal controls (OR: 3.14, *p* = 0.001), with higher expression in patients with dysplasia compared to those without dysplasia (OR: 10.76, *p* < 0.001). Although less pronounced, a significant difference in p53 expression was observed between UC cancer patients and patients with dysplasia (OR: 1.69, *p* = 0.035). Phosphoinositide 3-kinase (PI3K), a key signaling mediator in tumorigenesis, enhances cell migration, proliferation, and survival. Colon biopsies from UC and CAC patients revealed PI3K pathway activation, particularly involving the IL23R *rs10889677* variant (c.309C>A), which was highly prevalent. Alterations in interleukin-12 receptor beta 1 (IL12Rβ1) were also detected in 20% of samples (48, 49). Other inflammation-driven carcinogenic pathways implicated in UC include Toll-like receptors, cyclooxygenases, carbonic anhydrases 2/9, and microRNAs (miRNAs), all contributing to neoplastic transformation (50–53). These findings have been obtained from clinical studies.

Regulatory T cells (Tregs) suppress excessive immune activation and self-inflicted tissue injury (54). However, Tregs exhibit plasticity, converting into Th17 cells that exacerbate inflammation. In UC, Tregs can paradoxically produce IL-1 and IL-6, serving as effector cells that contribute to UC and CAC pathogenesis (55, 56). Another mechanism involves neutrophil extracellular traps (NETs), formed via NETosis—a distinct cell death pathway triggered by pathogens, immune complexes, and ROS. This process involves the activation of NADPH oxidase, myeloperoxidase (MPO), and neutrophil elastase (NE), leading to chromatin decondensation and NET formation. UC patients show elevated levels of peptidyl arginine deiminase 4 (PAD4), MPO, and NE in the colonic mucosa, suggesting the important role of NETs in UC-related inflammation (57). Additionally, autophagy and macrophage-mediated immune regulation have been linked to the transition from chronic colitis to colon cancer (58).

Gut microbiota alterations contribute significantly to colitis-associated carcinogenesis. Probiotic modulation using *Clostridium butyricum* (CB) and *Akkermansia muciniphila* (AKK) reduced weight loss in a dextran sodium sulfate (DSS)-induced IBD mouse model. Furthermore, in the azoxymethane (AOM)/DSS-induced CAC mouse model, the combined probiotic treatment reduced tumor burden and Ki67 expression (a proliferation marker). Treatment also increased Caspase-3 activity (an apoptosis marker) and decreased CD8+ T cell and macrophage infiltration, enhancing anti-PD-L1 therapy efficacy (59). Xu et al. (60) identified *Achromobacter pulmonis* (*A. pulmonis*), isolated from mesenteric adipose tissue of CD patients, as a colitis-aggravating bacterium in mice. Its type III secretion system (T3SS) gene cluster induced macrophage and epithelial cell necrosis via the effector protein AopD, independent of caspase activation. Clinical studies have shown similar findings. Fecal samples from CD patients showed a significant abundance of *Achromobacter* and T3SS core genes compared to healthy individuals and patients with UC/CRC. *Saussurea costus* (SC) also showed therapeutic potential in a UC mouse model, alleviating weight loss, fecal occult blood, and colon shortening. SC restored colonic architecture, reduced inflammation, and modulated the gut microbiota, decreasing the abundance of harmful bacteria, such as *Proteobacteria*, and increasing beneficial bacteria, including *Lactobacillus* (lactic acid bacteria) and *Firmicutes*. This shift in microbial community structure suggested that the therapeutic effects of SC may be partly attributed to its ability to restore gut microbiota homeostasis (61).

### Carcinogenic effects of immunosuppressive therapy

Immunomodulators (e.g., azathioprine [AZA], 6-mercaptopurine) and biologics, particularly TNF-α inhibitors, are cornerstone treatments for pediatric IBD (62–64). However, prolonged use of these medications may increase the risk of malignant tumors (**Figure 2**). Several results have been obtained from clinical studies.

AZA, an imidazole derivative of 6-mercaptopurine, suppresses nucleic acid biosynthesis and cell proliferation, potentially causing DNA damage. It is widely used for long-term IBD maintenance therapy, as it reduces disease activity, steroid dependence, relapse rates, and postoperative recurrence (65–67). However, the association between long-term AZA use and cancer risk remains debated. The following studies link AZA to fatal malignancies, including colorectal, skin, and hematologic cancers (68). A meta-analysis of 18 studies involving adult populations found that AZA significantly increases lymphoma risk, particularly in current users, with males having a higher risk than females. Thus, due caution should be exercised during long-term AZA treatment in elderly patients (>50 years) and young males (<30 years). Notably, 56% of lymphoma cases were EBV-positive, implicating immunosuppression-induced EBV reactivation as a possible mechanism (69). In contrast, a single-center retrospective study of 1,374 pediatric and young adult IBD patients identified only two lymphoma cases over 6,624 patient-years, both in males treated with thiopurines, but not biologics. No significant increase in lymphoma risk was observed in the subgroup of pediatric patients treated with thiopurine drugs (70). To some extent, lymphoma risk may reflect underlying IBD severity, with treatment benefits potentially outweighing the disease risks. Another meta-analysis of 27 studies (95,397 patients) found that AZA use was associated with a decreased risk of CRC. The analysis revealed a significant risk reduction, with case-control studies showing a 51% decrease in risk, and cohort studies revealing a slight 4% risk reduction. These data are derived from adult populations. Notably, AZA exhibited a chemopreventive effect in high-risk CRC patients with a longer disease course (>8 years). However, no protective effect was observed in patients with extensive colitis or concomitant PSC (71). Nonetheless, another meta-analysis revealed that AZA did not prevent progression from mild atypical dysplasia to severe atypical dysplasia or CRC in IBD patients (72). Since most of the present evidence is based on studies involving adult cohorts, more robust research is required to better characterize the carcinogenic effects of thiopurine drugs in pediatric populations.

TNF-α-inhibitors, such as infliximab and adalimumab, have revolutionized IBD treatment, enabling the achievement of clinical remission in patients with CD and UC (73, 74). These drugs neutralize pro-inflammatory TNF-α molecules, modulate intestinal immune responses, and promote mucosal healing (75). However, their long-term safety—particularly the potential malignancy risk in pediatric patients—remains a concern. A 2008 U.S. FDA report documented malignancies in children who received anti-TNF therapy for immune-related diseases (including IBD), prompting reduced AZA use in pediatric patients. Among 31 reported pediatric malignancy cases post-infliximab, 24 involved IBD patients. Notably, nine developed fatal HSTCL, while others developed NHL, Hodgkin lymphoma, or leukemia. Most IBD patients (20 of 24 patients) had received thiopurines; only four received methotrexate in combination (76). In contrast, basic research suggests that infliximab may reduce cancer risk. In a DSS-induced colitis mouse model, early intervention with infliximab significantly reduced CRC incidence from 75–80% to 16.7% (77). Supporting this, a 20-year U.S. cohort study (1999–2020) found that TNF antagonist use was associated with a significantly lower risk of CRC in IBD patients (adjusted OR: 0.69 for CD; 0.78 for UC) (78). A recent meta-analysis of 55 studies (57,518 patients) found no significant increase in overall cancer risk with TNF-α inhibitors—including adalimumab, infliximab, etanercept, certolizumab, and golimumab—in both interventional and observational studies (79). Although a trend toward increased risk emerged with long-term follow-up, most studies had short follow-up periods, highlighting the need for high-quality, long-term prospective studies to better assess malignancy risk and optimize adverse event monitoring (79).

### Carcinogenic effects of diet and the environment

Environmental factors, such as diet, lifestyle, and air pollution, have also been implicated in the development of IBD (**Figure 2**). Long-term high-fat diet (HFD) consumption disrupts intestinal barrier homeostasis in mice, accelerating CRC progression by promoting a chronic inflammatory microenvironment (80). HFD-induced inflammation not only drives malignant transformation but may also create a self-sustaining pro-cancer loop. Animal experiments have demonstrated that prolonged HFD exposure induces low-grade chronic intestinal inflammation, impairs mucosal barrier function, and increases IBD risk (81). Epidemiological data further support a positive correlation between excessive fat intake and CAC incidence. Experimental models to elucidate the molecular pathways linking diet and CAC remain limited. Activation of the STAT3 transcription factor plays a crucial role in CAC by upregulating anti-apoptotic proteins like Bcl-2, correlating with the clinical and pathological features of CAC (82). Clinical studies have revealed increased levels of phosphorylated STAT3 (pSTAT3) in the intestinal epithelial cells of patients with CAC (83). While the mechanisms by which HFD exacerbates CRC via STAT3 remain unclear, cross-disease evidence suggests STAT3 signaling is central to HFD-induced tumorigenesis. Specifically, the amplification of STAT3-mediated pro-cancer signaling cascades warrants further exploration. Furthermore, HFDs contribute to CAC by altering gut microbiota’s butyrate metabolism. Research has demonstrated that HFDs reduce the abundance of butyrate-producing bacteria (*e.g.*, *Clostridiaceae*), and lower intestinal butyrate levels, correlating with increased polyp formation (*p* = 0.007) and reduced ZO-1 expression (*p* = 0.004), a marker of epithelial barrier integrity. Notably, these carcinogenic effects were reversed by broad-spectrum antibiotics or butyrate supplementation, suggesting the microbiota’s role in CAC pathogenesis and supporting dietary or microbial interventions in high-risk IBD patients (84). A recent preclinical study demonstrated the anti-cancer properties of *Bacteroides plebeius*, a gut symbiont that degrades algal polysaccharides (85). In AOM/DSS-induced CRC mice, oral administration of *B. plebeius* reduced tumor number and size by 50% (*p* < 0.01), with less inflammatory infiltration in colon tissues. The proposed mechanism involves modulation of gut microbiota (increasing beneficial bacteria and decreasing pathogenic bacteria) and increased production of anti-cancer metabolites (*e.g.*, propionate and ursodeoxycholic acid [UDCA]) (85).

The increasing incidence of IBD in newly industrialized countries is linked to environmental factors, particularly air pollution. Mendelian randomization analysis identified PM2.5 as a significant UC risk factor (86). A UK Bio-bank study of 453,199 individuals found that increased exposure to nitrogen oxides (Nox), nitrogen dioxide (NO_2_), PM2.5, and a composite pollution score increased UC risk by 20–26% per each interquartile range (IQR) increment, with stronger risk in populations with high genetic risk, unhealthy diets, smoking, obesity, or alcohol use. Epigenetic analysis revealed significant associations between UC risk and methylation changes in the *CXCR2* gene (chemokine receptor), and the MHC III region (*e.g.*, AGPAT1 and DDAH2). These changes were validated in colonic tissues and correlated with gene expression regulation (87). The latter two genes play a crucial role in colitis and CAC (88).

## Screening and prevention strategies

### Colorectal cancer surveillance

**Figure 3:**
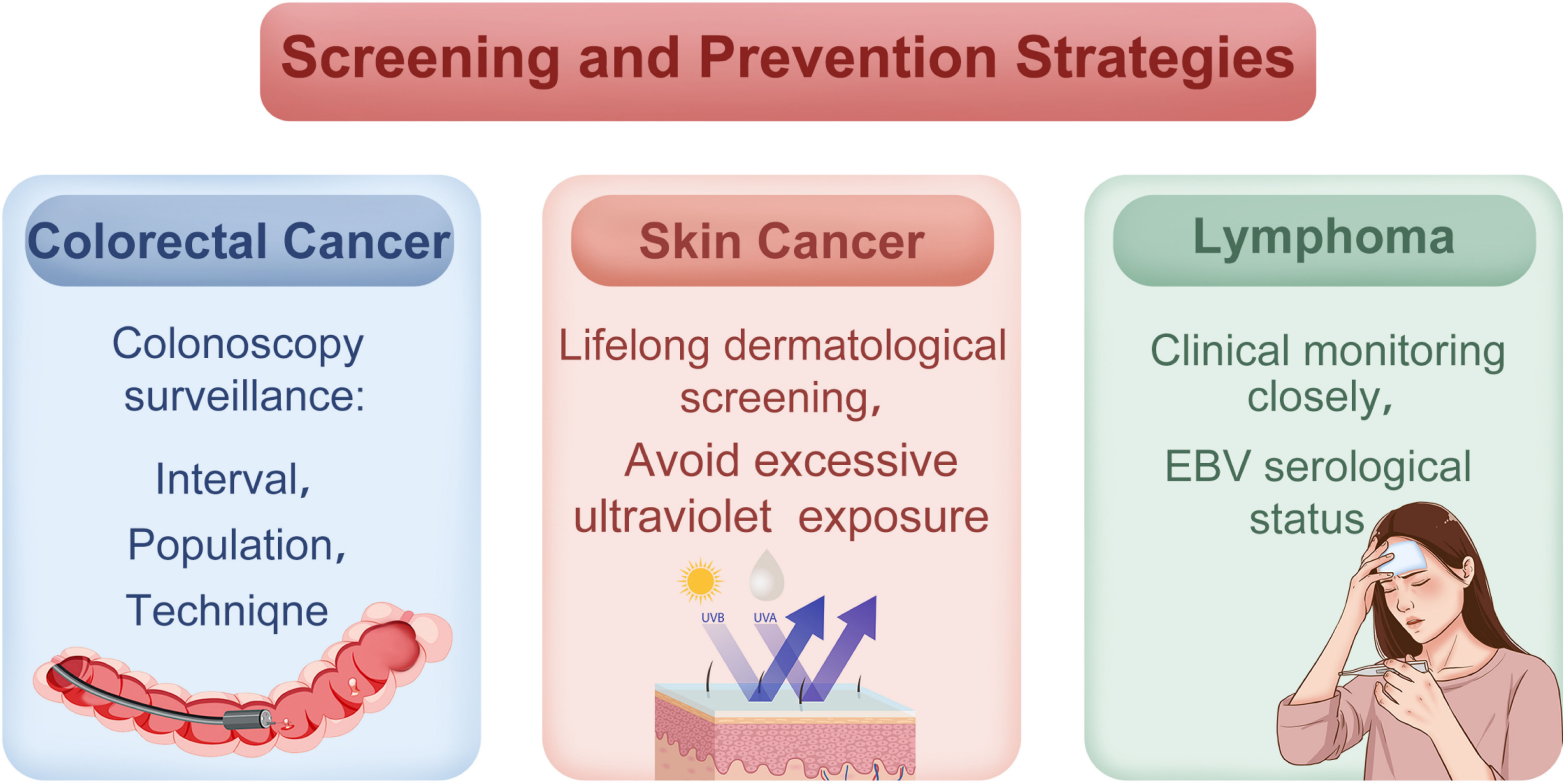
Cancer screening and prevention strategies in pediatric inflammatory bowel disease (IBD). Surveillance strategies include early colonoscopic monitoring, regular dermatological evaluations, and clinical monitoring for lymphoma-related symptoms in high-risk pediatric IBD patients.

The incidence of dysplasia and CRC in pediatric patients with UC has been underreported, resulting in the lack of data to inform a defined CRC screening protocol. Moreover, discrepancies exist in screening recommendations among various guidelines. The ECCO-ESPGHAN guidelines recommend initiating endoscopic surveillance at 8-10 years after disease onset, based on individual risk factors (89), using chromoendoscopy performed by experienced gastroenterologists. While optimal surveillance intervals remain undefined, ECCO-ESPGHAN recommends universal screening at 10 years after disease onset, whereas Dutch guidelines recommend screening only in children with extensive colitis (89, 90). Due to the scarcity of pediatric data, CRC surveillance guidelines for children with UC are extrapolated from adult studies. In adults, routine surveillance is not recommended for ulcerative proctitis, given the comparable CRC risk to the general population (91, 92). However, pediatric guidelines remain inconsistent due to limited evidence. PSC is an established independent risk factor for dysplasia and CRC in children with UC. Consequently, both pediatric and adult UC guidelines recommend annual or biennial surveillance colonoscopies starting from the diagnosis of PSC (**Figure 3**) (89–91).

Chromoendoscopy is the preferred surveillance method per guidelines, but limited access and expertise may hinder its use. As alternatives, the European guidelines recommend high-definition or white-light endoscopy combined with random biopsies as a suitable alternative. Furthermore, the Dutch adult IBD guidelines recommend white-light endoscopy with targeted biopsies (89, 91). Given technical limitations in pediatric settings, collaboration with experienced gastroenterologists is recommended. For high-risk groups, such as those with VEO-IBD or concurrent PSC, earlier and more frequent surveillance may be warranted.

### Skin cancer surveillance

Given the elevated risk of skin cancer in pediatric IBD patients, especially those who have received AZA, anti-TNF therapies, or combination therapies, some researchers have recommended lifelong dermatological screening. Given the scarcity of data on the pediatric population, adult guidelines are used as a reference. The primary focus should be screening for basal cell carcinoma, melanoma, and non-melanoma skin cancers, with emphasis on ultraviolet (UV) protection (e.g., sunscreen use, minimizing UV exposure) (**Figure 3**) (21). Danish researchers suggest incorporating skin cancer surveillance into standard care, particularly for male patients. However, the optimal frequency and methods for screening remain undefined (22, 33).

### Lymphoma surveillance

Adolescent males and patients on multiple immunosuppressants (*e.g.*, AZA + anti-TNF) require close monitoring for lymphoma. Due to limited data on children, guidelines for adult populations are referenced. Persistent fever, lymphadenopathy, hepatosplenomegaly, or cytopenia warrant evaluation for lymphoma, with the diagnosis confirmed through imaging (*e.g.*, computed tomography [CT]) and bone marrow biopsy (**Figure 3**) (27). EBV serology (IgM/IgG) should be assessed before and during treatment, as AZA may heighten lymphoma risk in EBV-positive patients (20, 26). For EBV-negative patients, EBV screening is recommended before initiating immunosuppressive therapy to assess lymphoma risk (26). Although no standardized protocol exists, regular clinical assessments are recommended every 6-12 months, with shorter intervals for high-risk groups.

## Conclusion

Children with IBD have a significantly increased risk of developing malignancies due to chronic inflammation and immunosuppressive therapies. The incidence of cancer in this population is trending towards a younger onset. Common cancers include CRC (highest incidence, especially with PSC or extensive colitis), small bowel cancer (mainly in CD), lymphoma (notably in males and AZA users), cholangiocarcinoma/hepatocellular carcinoma (linked to PSC), and skin cancers (primarily basal cell carcinoma and malignant melanoma). The pathogenesis of cancer in IBD involves genetic variants induced by chronic inflammation, carcinogenic effects of immunosuppressive agents, and environmental factors (e.g., HFD and air pollution). Chronic inflammation drives tumorigenesis through oxidative stress, cytokine network imbalance, and gut microbiota dysbiosis, while immunomodulatory therapy, though effective in controlling inflammation, may increase the risk of lymphoma and other cancers. Early surveillance—colonoscopy, dermatologic exams, and lymphoma screening—is essential for high-risk groups, such as those with early-onset IBD or coexisting PSC. Most existing data are from small Western cohorts, highlighting the need for larger, prospective studies to refine cancer prevention and treatment strategies tailored to children with IBD.
